# Dendritic cell maturation assay for non-clinical immunogenicity risk assessment: best practices recommended by the European Immunogenicity Platform

**DOI:** 10.3389/fimmu.2025.1704045

**Published:** 2025-11-19

**Authors:** Chloé Ackaert, Bruno Gonzalez-Nolasco, Marc Rosenbaum, Mercedes Perez-Olivares, Michael Gutknecht, Axel Ducret, Anette Christine Karle

**Affiliations:** 1 IQVIA Laboratories, In vitro Immunology (ImmunXperts SA), Gosselies, Belgium; 2 Lonza: Early Development Services, Lonza Biologics Inc., Cambridge, MA, United States; 3 Sandoz: Clinical Bioanalytics, Global Clinical Development, Hexal AG (a Sandoz company), Holzkirchen, Germany; 4 Abzena: Bioassay Department, Abzena Ltd, Cambridge, United Kingdom; 5 Novartis: Immunogenicity and Mechanistic Immunology, Biomedical Research, Novartis Pharma AG, Basel, Switzerland; 6 Roche: Pharmaceutical Sciences, Roche Innovation Center Basel, Basel, Switzerland

**Keywords:** dendritic cells, maturation assay, immunogenicity, risk assessment, adjuvanticity, innate immunity

## Abstract

Early assessment and mitigation of non-clinical immunogenicity risk during early drug development is key for the development of safe and efficacious therapeutics. The dendritic cell (DC) maturation assay, one of the non-clinical immunogenicity risk assessment tools used in the drug development pipeline, investigates the ability of a test article to induce the maturation of immature monocyte-derived DCs, serving as an indicator of factors that may initiate an innate immune response and contribute to an adaptive immune response. These factors can be either intrinsic to the therapeutic’s mechanism of action and structure, or extrinsic from the final drug product, such as formulation components or contamination with host cell proteins or other impurities. Due to the nature of the assay, key parameters such as cell source, cell culture conditions, reagents, and assay-specific defined criteria for baseline response and positivity can differ amongst laboratories. In this manuscript, the specifics of this assay are discussed, key quality criteria for robustness are described, and the selection of appropriate controls to enable meaningful data interpretation are presented. The aim of conducting the DC maturation assay using best practices is to improve the assay to be fit-for-purpose and to facilitate comparability across projects and between laboratories.

## 1 Introduction

Dendritic cells (DCs) are professional antigen-presenting cells (APCs) serving as a link between the innate and adaptive immune system by recognizing pathogenic stimuli and presenting antigen-derived peptides to T cells. They undergo morphological and functional changes broadly categorized into immature (iDCs) and mature (mDCs) stages. DCs are classified into several subsets, including conventional type 1 (cDC1), type 2 (cDC2), and plasmacytoid dendritic cells (pDCs)(1). Additionally, under inflammatory conditions, monocytes can differentiate into monocyte-derived DCs (moDCs), which share functional similarities with conventional DCs and are often used as a tool in *in-vitro* assays due to their accessibility.

**Figure 1:**
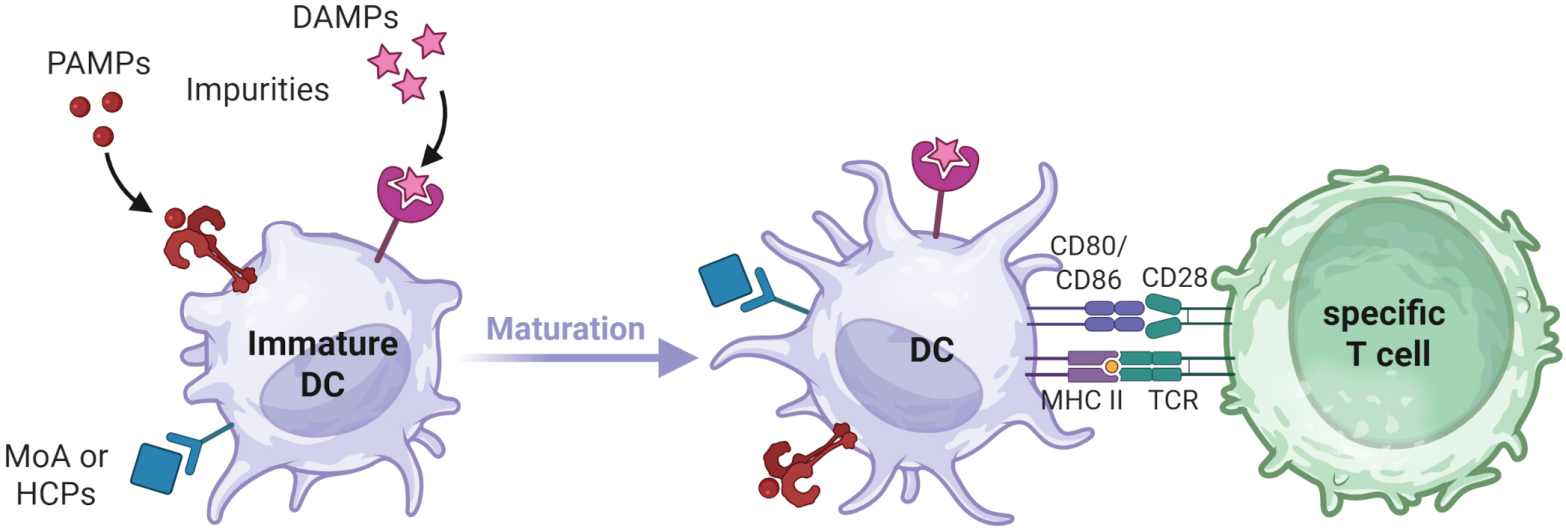
DC maturation process. Upon encountering PAMPs, DAMPs, MOA related stimuli, or impurities that trigger corresponding pathways, immature DCs undergo maturation. This process leads to morphological changes, increased processing and presentation of internalized antigens via HLA class II molecules and the upregulation of costimulatory molecules on the DC surface. T cell activation requires recognition of cognate antigen-derived peptides on HLA class II molecules (Signal 1) as well as interaction with costimulatory molecules on mature DCs (Signal 2). Created in BioRender. Karle, A. (2025) https://BioRender.com/fmrjm0v .

DCs internalize and process antigens, followed by the presentation of antigen-derived peptides on their surface to T cells via human leukocyte antigen (HLA) class II molecules. These HLA:peptide complexes can serve as epitopes if they are specifically recognized by the T cell receptors (TCR) of CD4+ T cells, which is a prerequisite for the induction of a primary adaptive T cell response, commonly referred to as signal 1. In addition, naïve T cells also require a signal 2 for activation, which is provided by CD28 binding to B7 family molecules such as CD80 or CD86 on the surface of DCs (**Figure 1**). The upregulation of B7 molecules and other costimulatory cell surface receptors, including CD83 and CD40, depends on the DC’s maturation state. This maturation process can be triggered during infection and/or inflammation by the recognition of pathogen-associated molecular patterns (PAMPs) or damage-associated molecular patterns (DAMPs) through pattern recognition receptors (PRRs).

In pharmaceutical development, biotherapeutics continue to address unmet patient needs through conventional and more innovative modalities, including peptides, antibodies, antibody fragments, nucleic acids, and cell and gene therapies. A major challenge associated with these biotherapeutics is their capacity to elicit unwanted immune responses, which can alter their safety and efficacy (1). As therapeutic modalities increase in structural and functional complexity, they increasingly diverge from endogenous human molecules, potentially elevating both the incidence and mechanistic diversity of immune responses.

A series of preclinical tools have been utilized as part of the drug development process to assess the immunogenicity potential of these therapeutics (2). The majority of these *in silico* and *in vitro* assays are designed to investigate downstream events in the adaptive immune response, particularly focusing on the identification of T-cell epitopes. For instance, the major histocompatibility complex (MHC)-associated peptide proteomics (MAPPs) assay identifies naturally processed and presented MHC-peptide complexes generated after the proteolytic processing of the therapeutic protein by APCs (3, 4). Additionally, various assays provide relative measurements of T-cell activation and proliferation in diverse experimental setups (5).

In addition to assessing adaptive immune responses, the non-clinical immunogenicity risk assessment toolkit includes assays designed to evaluate a therapeutic’s potential to trigger innate immune activation. In this manuscript, the focus is on the DC maturation assay, however, there are other types of assays to assess the innate immune activation. *In-vitro* generation of moDCs have been a useful tool for studying key events in the immunogenicity cycle, including antigen uptake and presentation, as well as delivering costimulatory signals for T cell engagement and polarization (5). The DC maturation assay leverages both phenotypic changes and cytokine production by moDCs upon exposure to a therapeutic of interest to assess its adjuvanticity, which may contribute to the drug’s immunogenicity potential. The DC maturation assay is a nonclinical immunogenicity risk assessment tool for the analysis of product-related risk factors that have the potential to induce the maturation of DCs and thereby inform the contribution to the risk of developing anti-drug antibodies (ADA). Instead of immunogenicity risk, immunogenicity potential is also a common term used in the context of these assays. It is important to note that DC maturation assessments do not inform the potential clinical consequences for the patient and its potential association with ADA development.

In a joint effort, the European Immunogenicity Platform Non-Clinical Immunogenicity Risk Assessment working group (EIP-NCIRA) has attempted to harmonize antigenicity assays (2). A comparison of current DC maturation assay approaches across companies revealed notable differences based on historical assay set up, rendering complete protocol standardization unrealistic. Several detailed example protocols can be found for example in Morgan et al. (6), Siegel et al. (7) and Wickramarachchi et al. (8). Rather than protocol standardization, our aim is to provide best practices for conducting DC maturation assays that will improve assay robustness and comparability across projects and between laboratories, through which we aim at addressing the importance of context-of-use validation and the challenges of standardization reported previously in regulatory and review publications (2, 9–11). To this end, we specify the purpose/objective of a DC maturation assay and highlight certain key elements and steps in the workflow that most strongly impact the outcome of the assay to enable comparability of results. These key features include the source of peripheral blood mononuclear cells (PBMCs), PBMC quality control (QC), the cell purification method for moDC maturation assays, as well as cell culture conditions for moDC differentiation. We also provide recommendations for the QC of iDCs, the loading of iDCs and respective controls, and the QC and assessment of mature DCs. Thus, we recommend a standardized set of controls and minimum quality features across various readouts, while accommodating unique optimizations implemented by each laboratory. In addition, we address crucial parameters in assay performance qualification and for the interpretation of results.

## 2 Purpose/objective of the DC maturation assay

As a component of the preclinical toolkit, the DC maturation assay can be integrated into the drug development pipeline as a component of the preclinical immunogenicity risk assessment strategy. The DC maturation assay assesses the ability of a test article to induce the maturation of immature moDCs, serving as an indicator of factors that may initiate an innate immune response and contribute to an adaptive immune response. These factors can be intrinsic, linked to the therapeutic’s mechanism of action, or extrinsic, associated from the final drug product’s critical quality attributes (CQAs) and formulation components. In some cases, drug products are capable of triggering the maturation of DCs depending on the mode of action (MOA) and structure of the biotherapeutic itself (12), the route of internalization (13), the presence of aggregates (14), contamination with host cell proteins (15, 16) or other impurities (17), or formulation components. The three last aspects can induce effects similar to PAMPs and DAMPs. The induced adjuvanticity can be problematic stand-alone in certain cases. In addition, adjuvanticity can contribute to the development of immunogenicity, and understanding the root cause of the DC maturation based on impurities, MOA, or structure of the biotherapeutic may help to identify solutions to reduce the adjuvanticity-related immunogenicity potential of a drug.

The DC assay enables the comparison and ranking of different test candidates in terms of their ability to induce DC maturation. These different test candidates may be variants from molecules in development processes, production batch changes assessed against one another, or molecules assessed against a similar clinically validated benchmark. Results may be useful to redesign the biotherapeutic, to change the formulation buffer, or to minimize impurities to decrease DC activation by the drug product.

This assay has also proven valuable in assessing the mechanistic impact of aggregated species within therapeutic products (18), which is one of the best studied factors known to influence immunogenicity of biotherapeutics. Numerous studies have been conducted to identify the mechanism by which aggregates may enhance the immunogenic potential of the drug (6, 14, 19). Joubert et al. elucidated four mechanisms by which aggregates could enhance immune responses, the first two of which can be studied via DC maturation assays: 1) recognition of repeated motifs mimicking PAMPs via PRRs on APCs; 2) interaction with Fc receptors triggering increased antigen uptake and potentially leading to increased activation; 3) activation of the complement system, and 4) enhanced presentation of T-cell epitopes (18).

Besides screening approaches for candidate selection, under specific conditions, the assay can be utilized for mechanistic studies, investigating stimulatory effects mediated by target engagement on DCs, candidate payload effects, impact of CQAs or formulation components (20). It is important to note that the absence of observed DC maturation in this assay does not imply the absence of T cell epitopes in the therapeutic product. To address this challenge, additional assays that evaluate other elements of the immune response such as MAPPs and T cell assays are commonly incorporated. Therefore, the DC maturation assay can be used alongside these other preclinical immunogenicity assays to assess different aspects of the immune cascade.

Design components of the DC maturation assay play a role in the interpretation of the results. Section 2 explores these parameters in the context of assay harmonization, highlighting key variables such as test article concentration, and quality of the test articles themselves. Notably, the CQAs of a biotherapeutic greatly depend on the developmental stage, with early-stage materials often differing substantially from those in later stages of development, which apply refined purification and formulation processes. Therefore, the objective for testing the biotherapeutic might differ across different stages of drug development. In early stages, the ability of the drug itself to induce DC maturation is in focus, while in late stages the mechanistic impact of formulations and CQAs on DC maturation may become relevant. In this context, the scope of the DC maturation assay in comparison to an Innate Immune Response Modulating Impurities (IIRMI) assay may be of interest. A DC maturation assay can provide valuable information on the factors related to the biotherapeutic itself and its capacity to induce maturation of DCs as a necessary step to recruit other immune cells and stimulate an adaptive immune response. In contrast, the IIRMI assay is a cytokine release assay performed on whole blood or PBMCs (21, 22). Its key advantages include reduced cell handling, shorter assay duration, and the ability to assess the immediate immune response across all PBMCs. However, since the immune cells are not purified beforehand, a positive response in the IIRMI assay lacks specificity and cannot be attributed to a particular cell subset. Nevertheless, it provides an overall idea of the drug product’s potential to induce innate immune activation. An IIRMI assay can be used quite early in development to ensure that investigational products used in preclinical testing are free from impurities that would shift the results. It is also valuable at a later stage of development, particularly for generic peptides in their final formulation, to demonstrate equivalence between a generic peptide and its respective reference-listed drug. This latter application has led to the IIRMI assay being frequently used in a regulatory context for Abbreviated New Drug Application (ANDA) submissions. In contrast, while the DC maturation assay can be included in regulatory submissions, it is more frequently used internally, supporting the selection and qualification of biotherapeutic candidates.

Besides the variables of the test products, the inclusion of appropriate controls, for which specifications are provided in section 2.5, is important to ensure the assay is fit-for-purpose. This aspect is detailed further in Section 3.

## 3 Key elements to control

### 3.1 Source of PBMCs and PMBC quality

High-quality PBMCs are a critical starting material for most *in vitro* non-clinical immunogenicity risk assessment assays. PBMCs can be obtained by sourcing and processing various blood products, including whole blood, leukopaks, or buffy coats. While standardized isolation protocols exist, laboratories often implement their own optimized procedures, based on blood source, available infrastructure/instrumentation, individual laboratory process needs, or connection with other types of assays, resulting in inter-laboratory variability. PBMCs may be used directly after isolation from fresh or cryopreserved material with different effects on cell viability and function. These operational variables, along with logistical factors such as sample shipment, storage conditions, and handling, can further influence the phenotypic and functional properties of the APCs used in DC activation assays, and present challenges to harmonization. However, the implementation of general QC recommendations for both the initial PBMC material and the final cells generated through these workflows provides a valuable opportunity to promote assay standardization. These considerations for PBMC isolation QC are valid across all *in vitro* assays and a summary of the key considerations from these guidelines that can help to determine donor exclusion criteria in *in vitro* assays is listed below:

o The nature of the initial blood product and the way it was processed can affect the quality of isolated PBMCs and should be considered when selecting donor material.
o The time from blood draw to PBMC isolation is a critical factor and should ideally not exceed six hours.
o When using cryopreserved cells, it is important to follow established standard operating procedures (SOPs) for both cryopreservation and thawing.
o QC can be performed on cryopreserved samples prior to assay execution, allowing for donor qualification. Conversely, freshly isolated PBMCs can be used immediately, avoiding stress from freeze/thaw cycles; however, clear criteria should be established to exclude suboptimal samples.
o The timing of blood draws can influence PBMC quality, as seasonal illnesses and other immunological stressors may affect immune cell populations and their homeostatic balance, even within the healthy donor pool.
o HLA typing is not required when using the DC maturation assay as a stand-alone approach. Notably, HLA data adds value when conducting sequential immunogenicity potential assessment studies across innate and adaptive immune endpoints using the same donor cohort.
o PBMC QC can encompass multiple parameters, including cell viability, phenotypic profiling, and functional testing. Common flow cytometry markers include viability dyes and fluorescently labeled antibodies to characterize key leukocyte subsets such as T cells, B cells, NK cells, monocytes, and DCs.
o Suggested donor exclusion criteria include PBMC viability <90%, a stimulation index (SI) <2 in response to a strong positive control [e.g., keyhole limpet hemocyanin (KLH), cytomegalovirus (CMV)], or post-thaw recovery <90%. Phenotypic data can also guide donor selection for moDC generation, giving preference to donors exhibiting high monocyte percentages.

### 3.2 Cell purification method

For moDC maturation assays, CD14+ monocytes need to be isolated from PBMCs and a variety of methods are available, each yielding distinct outcomes in terms of purity, recovery, and cellular functionality (23). In the context of non-clinical immunogenicity risk assessment assays, both magnetic-activated cell sorting (MACS) and plastic adherence-based enrichment techniques are commonly employed. Within this EIP-NCIRA working group, a preference was revealed for MACS via either positive or negative selection using various commercially available microbead and column systems. Although MACS is more costly than the adherence method, its ability to achieve higher purity, particularly through positive selection, was favored. However, it is worth noting that residual magnetic beads may be carried over downstream, potentially influencing later observations. Technologies enabling bead dissociation from target cells may provide a viable solution to this limitation. Nonetheless, both positive and negative MACS-based selection methods are generally regarded as reliable and capable of providing reproducible and high-quality results. Ultimately, a QC of the isolated CD14+ cells should be included as part of the workflow to ensure their viability (>90%) and purity (percent CD14+ >90%).

### 3.3 Cell culture conditions for moDC differentiation

Based on shared protocols from the companies involved in the EIP-NCIRA working group, we provide an overview of the cell culture conditions for moDC differentiation (**Table 1**). This shows where more flexibility is possible and what advantages and disadvantages are associated with the various options. Our internal survey continued to highlight how different groups have optimized cell handling and culturing. Given the remarkable plasticity of moDCs, methods should aim to minimize cellular stress by minimizing mechanical handling and preserving cell integrity, viability and phenotype.

**Table 1:** Cell culture components for differentiation of monocytes into moDCs.

Component	Industry-wide use	Considerations
Culture container	Plates (tissue culture treated surface/low binding), flasks (tissue culture treated surface), clinical-grade bags	Assay parameters may need to be re-optimized if different culture containers are used, as they can influence the phenotype and function of moDCs (24, 25).
Culture medium	RPMI total/with supplements or specific DC media from different suppliers	Assay needs to be optimized based on chosen medium.
Density of monocyte seeding	Seeding for differentiation (26): 0.5 to 2 x 10^6^ cells/mL	Seeding density is surface area dependent
Cytokines for differentiation of monocytes to iDCs	Both, IL-4 and GM-CSF (27)	Cytokines are available from different suppliers. Biological activity may differ across suppliers and lots. Titration is required for each lot to determine optimal assay concentrations.
Differentiation time	5–6 days	This timeframe has successfully been used across laboratories.
Medium and cytokine refreshment	Varies across industry. Protocols with and without medium addition/refreshment	No full medium exchange to avoid harvesting or disturbing cells Refreshment possible by partial medium exchange with cytokines.
Harvesting of iDCs	Pipetting with PBS (addition of EDTA can help improve detachment; gentle scraping has also been reported, and, if feasible, it is preferable to avoid mechanical stress. The effects of any detachment method should be carefully monitored); syringe extraction (bags); or kept in original container	Gentle handling of immature DCs, as mechanical stress may induce pre-maturation and potential false negative outcome when treated with drug; inclusion of a flow cytometric QC step to verify that the cell harvesting procedure has not inadvertently induced DC activation
Cell density of iDCs	1 x10^5^ to 6 x 10^5^ per test condition, although a higher range can be used as well	Optimal cell density may vary depending on the specific experimental aim
Incubation time of iDCs with test molecules	4 - 48h	optimal duration is project specific and might need to be optimized for each project

### 3.4 Recommendations for the QC of iDCs

iDCs should be properly differentiated, and not pre-activated, as pre-activated cells tend to be less efficient in antigen uptake. The following recommendations for the QC of iDCs based on viability and fluorescence-activated cell sorting (FACS) markers are suggested by this EIP-NCIRA working group. As the QC is based on flow cytometry analysis, good FACS practices should be applied, including titration of antibody clones for each parameter. A live/dead marker enables the assessment of viability of iDCs, which should be ideally >95% when gating on the iDC population. It is important to note that this metric can be influenced by the initial gating of the cell population to consider, with a looser gating including more dying cells. Therefore, a safe lower limit when excluding debris can be defined at 70%. iDCs should be negative for the monocyte marker CD14 and positive/high in differentiation markers CD11c, CD209. CD1a can be used as well and is expected to be medium to highly expressed in iDCs, but this can be donor and process dependent. Maturation markers such as CD80, CD83, CD86, CD40, HLA-DR (+ DP + DQ), (HLA ABC, as QC marker), CXCR4, should still be relatively low at this stage (28). Differentiation of cells should be confirmed via microscopy (29). Whereas monocytes are small and round, relatively smooth cells sticking to the plate, iDCs are more elongated cells with dendrites, and loosely adherent. These dendrites become even more pronounced upon maturation. Those that do not align with the phenotype and still show CD14 expression should be discarded, as this could imply incomplete differentiation. Depending on the aim of the DC maturation assay, the breadth of the FACS panel might be tailored: when run in conjunction with a MAPPs assay, a limited FACS panel may suffice, whereas a standalone application aimed at more in-depth characterization benefits from an extended panel.

An overview of the key markers is provided in **Table 2**, with indication of relative expression levels of the different markers at each stage: +, ++ and +++ are representing low, medium/high, high/very high relative expression levels.

**Table 2:** DC markers at the stage of monocytes, iDCs and mDCs.

Marker	Function	Monocytes	iDCs	mDCs
CD14	LPS-induced Macrophage activation	+++	-/+	–
CD11c	Widely known as DC marker with various roles (30)		+++	+++
CD1a	Glycoprotein presenting lipid Ags to T cells (31)	–	+	+
CD80	Co-stimulation for T cell activation	–	+	++
CD83	Lymphocyte activation	–	-/+	++(+)
CD86	Co-stimulation for T cell activation	–	++	+++
HLA-DR	Antigen Presentation class II	+	++	+++
HLA ABC	Antigen Presentation class I	QC marker only		
CD209	DC-T cell interactions, DC migration	-/+	+++	++
CD40	Co-stimulation for T and B cell activation	+	+	+++
CXCR4	Chemokine receptor with multiple roles (32)	–	-/+	+++

### 3.5 Recommendations for culture and loading of iDCs

This section outlines critical parameters and considerations for the efficient and standardized loading of iDCs with test molecules, aiming to minimize variability and enhance assay reproducibility, also summarized in **Table 1**.

o Culture medium: The choice of medium may influence iDCs viability and function and should align with the intended application. Commonly used media include RPMI 1640 (with or without supplements) and DC culture medium from different suppliers. With regards to the addition of serum to the culture medium, different laboratories are using both serum-free and fetal calf serum (FCS) or human AB serum containing media. The latter need a rigorous QC for each lot and batches, so well controlled and characterized lots/batches are used (33–35).
o Cell density: Two main cell counting strategies are used in the field: counting at the monocyte state prior to differentiation into moDCs, and performing an additional count after differentiation at the iDC stage. The latter approach allows for more consistent cell numbers across experiments but requires cell harvesting, which may induce iDCs pre-activation. The major advantage of counting the cells at the iDC stage is that for each condition within the same donor the same drug concentration and the same FACS antibody concentration is used, allowing for a consistent relative comparison between conditions. Section 2.3 provides information about industry-standard cell densities for monocyte differentiation. For the maturation phase, optimal cell density may vary depending on the specific experimental aim and should be determined empirically; however, a range of 1 x 10^5^–6 x 10^5^ per test condition is commonly recommended, although a higher range can be used as well.
o Test molecule concentration: Loading concentrations vary across protocols, with some laboratories using fixed concentrations, such as 0.3 µM or 0.4 µM, while others employ a range of concentrations to evaluate dose-responses and address assay sensitivity. On that note, it is especially important to assess a range of concentrations during assay development and to ensure that the sensitivity controls (see control section below) are used at the same concentrations to identify the optimal experimental conditions.
o Incubation time: The incubation period for iDC loading typically ranges from 4 to 48 hours, but the optimal duration should be selected based on the characteristics of the test molecule and the specific question being addressed (i.e. early vs late responses).

### 3.6 Assay controls

Controls must be appropriately selected and validated based on the specific assay context to ensure reliability and reproducibility. These include:

o background/baseline controls, which serve as negative references;
 These background controls can be unstimulated cells or cells treated with formulation buffer from the drug product (placebo). Their inclusion enables monitoring of the immature state of the cells throughout the assay and provides a reference for any response elicited by the buffer alone.
o cell functionality controls, which serve as technical controls for each component of the assay;
 These controls are included to monitor the functionality of the cells in the assay.
 Commonly used cell functionality controls across industry within this working group are lipopolysaccharide (LPS) (a TLR-4 ligand), Poly I:C and R848 (ligands for TLR3 and TLR7/8), MPLA and IFN-γ, KLH or a cytokine maturation cocktail.
 Special consideration must be given to the source and lot of LPS used, as different variants may elicit different responses (36) and influence the expression of phenotypic markers such as CD14. On mature DCs, CD14 expression is low to absent, except when LPS is used as a stimulus (as a control or as an impurity in the test product), since it leads to increased CD14 expression. (37)
o sensitivity controls are included to evaluate the assay’s sensitivity, meaning they can assess the ability of the assay to detect responses to test articles and establish its dynamic range. These controls should be biologically relevant molecules from which the assay’s response profile is known, and ideally, they should be modality specific.
 Examples of sensitivity controls for biotherapeutics are bevacizumab (Avastin) as low DC maturation control, and ATR-107 (an anti-IL21R mAb) (12) as high DC maturation control. To differentiate undetermined activation mechanisms from biological engagement (i.e., target expression on the DCs), a suitable control (known in clinic) can be added if available and of additional value to the approach.

Together, cell functionality and sensitivity controls provide a framework to judge assay/experiment quality and enable proper interpretation. Cell functionality controls allow for the evaluation of the assay performance and whether a donor meets the predefined inclusion criteria, whereas sensitivity controls allow for the evaluation of the assay sensitivity, enabling meaningful interpretation of results. In this context, sensitivity controls play a particularly important role, as they are critical for evaluating a positive response to the test article.

Performance of used control materials should be regularly monitored, and in-house qualifications are essential to address lot-to-lot and supplier variability. If a clinical-grade drug product is unavailable for use as sensitivity controls, drug substances produced alternatively require thorough quality assessment, ensuring minimal content of endotoxins, aggregation/degradation and other impurities. For example, the choice of expression host cell line can significantly influence post-translational modifications, potentially altering the biological activity of the sensitivity control despite identical amino acid sequence to the clinical-grade one. In addition, host cell proteins and other contaminations may modulate maturation capacity of the control molecule (15, 16).

### 3.7 Recommendations for the QC and assessment of mature DCs

DCs stimulated with cell functionality controls should be properly matured. The following section provides recommendations for the QC of moDCs based on viability assessment and FACS markers.

A live/dead viability dye should be included to assess cell viability. Cell gating for phenotypic characterization can be performed using markers such as CD11c, CD1a and/or CD209, with CD14 optionally included as an additional QC marker as it should remain negative unless LPS was used for maturation. Maturation markers should be measured to evaluate the extent of maturation induced by cell functionality controls, confirming maturation capacity of controls in the performed assay: CD80, CD83, CD86, CD40, HLA-DR (DP, DQ), HLA ABC, CXCR4. Marker selection depends on the research question and the purpose of the assay. A minimal QC panel should include a viability dye and CD80 or CD83 or CD86 and HLA-DR.

The specifics of an optimal DC maturation profile are based on laboratory-specific cell functionality controls. While the maturation profile achieved by a functionality control would define the maximal maturation state of the DCs, sensitivity controls shape different maturation profiles, ranging from no or minimal maturation for negative sensitivity controls to partial upregulation of specific markers for positive sensitivity controls.

Exclusion criteria at mature state would comprise 1) viability < 80%, 2) failure to upregulate a minimal panel of markers following stimulation with the functionality control, or 3) strong activation marker expression in the background/baseline control.

For those DCs that passed QC, several read-outs to measure maturation can be used. Flow cytometry is the most common read-out for the assessment of DC maturation by drugs across industry. Commonly assessed surface markers comprise CD80, CD83, CD86, CD40, HLA-DR (DP, DQ), HLA ABC, CXCR4. Marker selection depends on the research question and the purpose of the assay.

Although the main focus of this manuscript is on DC activation studies using DC activation markers as biomarkers, additional maturation readouts include 1) cytokine quantification in the supernatants of the DC cultures via different platforms: IL-1β, IL-6, IL-8, IL-12p40, TNF-α (note that cytokine stability must be validated before the analysis if supernatants are frozen), 2) mRNA expression analysis via PCR, and 3) cell signaling studies, as described by Xue et al. (12). In addition, a novel approach for assessing DC activation in combination with DC migration potential was described by Jarvi and Balu-lyer (28). Their study demonstrates how the migratory capacity of moDCs can be evaluated using a transwell assay and proposes this parameter as a mechanistic marker for immunogenicity screening. This is achieved by measuring intracellular expression of CXCR4, alongside activation markers CD40 and IL-12, following exposure to a concentration gradient of the therapeutic protein as well as chemokines CCL21 and CXCL12.

### 3.8 Establishing methodology and training for assay execution

During assay development, different options can be tested and optimized within each laboratory to make the assay as performant as possible in-house. Once the assay is qualified, the production-ready version is deployed along with an established SOP, which shall be followed for each run, as consistency of methodology is important for robust results. Future optimizations should undergo a similar process for deployment. Lastly, best practices for assay execution include documented staff training and assay performance tracking.

## 4 Assay performance qualification

Understanding the analytical performance of the DC activation assay is essential for data interpretation and drawing reliable conclusions. While traditional assay validation characteristics (38) should be considered during the experimental setup, the inherent complexity and variability of this primary cell-based assay present challenges to conducting a complete assay validation. A fit-for-purpose (FFP) validation approach (8) offers a practical framework to evaluate key assay parameters such as precision, sensitivity, specificity and robustness. The selection and investigation of the baseline response control, the cell functionality control, and sensitivity control become instrumental during initial assay setup. Together, the baseline response control and cell functionality control can be utilized to establish statistical thresholds, and to define donor acceptance criteria. These controls facilitate longitudinal assay performance monitoring, enabling the establishment of run-level acceptance criteria. If a systematic change over time is observed, a root cause analysis might be required. In this context, although the specific metrics monitored may differ between laboratories, the quality assurance strategy should be well defined and documented.

Sensitivity controls, which more closely resemble the test articles in nature and functionality, provide a comparison metric for interpreting the relative immunostimulatory potential of unknown samples. Moreover, they are useful to confirm an appropriate donor cohort size to determine the impact of a similar biotherapeutic on maturation.

Once there is clarity on the assay performance metrics specific to the experiment’s setup, it is helpful to establish a strategy to understand possible sources of variability as well as assay health over time.

This variability can be either biological or technical in nature. A portion of that variability can be controlled by ensuring consistent donor material sourcing and adherence to established SOPs for cell handling, reagent qualification, and instrument calibration. Apparent outliers in the dataset can be addressed using various statistical methods for replicate analysis. However, the impact of removing those outliers should also be evaluated, as their influence on the overall readout may be minimal depending on the central tendency metric used to derive the assay readout. (8)

## 5 Interpretation of results

Assay results should be interpreted within the assay settings specified in the assay performance qualification and the thresholds determined based on the selected baseline response, cell functionality and sensitivity controls. No specific limits for positivity are communicated here, as the specific values depend on the concentration of the viability dye, the antibody clone and its concentration, as well as the flow cytometry instrument and settings. The decision as to whether a response to the test article is considered positive should always be made in the context of the sensitivity controls used. If relevant to the project, these sensitivity controls should comprise differential DC maturation potentials, i.e. divergent immunogenicity profiles with low, medium and high known DC maturation potential. It is recommended to predefine the markers that need to be upregulated over the baseline response control signals for a response to be considered positive, ideally based on the sensitivity controls used. Similarly, where ratios are reported, a threshold for a positive response should be predefined based on the sensitivity controls used and will thus guide the decision whether the test article has adjuvant potential and could contribute to the immunogenicity of the test article. Note that large differences between donors are normal when working with human primary cells. Therefore, the most common way to overcome this inter-donor variation is to calculate fold changes over the untreated cells or background/baseline condition and use this ratio for data interpretation.

The number of replicates needed depends on the stage of assay development. In the early/set-up phase, duplicates or even triplicates might add value, with a preference for biological replicates (different samples/wells treated with the same condition) over technical replicates (multiple measurements of the same sample/well). Once the assay performance is qualified and the assay variability characterized, singlicate analysis might be sufficient.

The number of donors included for this assay within this working group ranges from 5 to 10 donors with a positive response to the cell functionality control.

Based on the defined fitness of the assay and the predefined criteria that would determine a positive response, each condition can be evaluated within each donor, and a general outcome can be obtained from a cross-donor evaluation for each condition.

From a statistical perspective, the approaches used within this working group comprise the calculation of the fold change over the baseline response control, a 2-way ANOVA and equivalence testing.

## 6 Discussion and future considerations

DC maturation assays are commonly used as a first-line *in vitro* assessment to investigate the adjuvanticity and immunogenicity potential of biotherapeutics by virtue of a small experimental footprint and the analytical ease of recording relevant activation signals. Concomitantly, one of the most common pitfalls related to conducting a DC maturation assay is the correct assignment of a potential signal to the test molecule. In early stages of drug development, molecules are not commonly available in their final formulation and purity (since the clinical-grade material has not yet been produced), while there might be an early need to assess the potential for immunogenicity. Therefore, as a general guideline, it is important to test the molecule at a high purity level. Moreover, buffers deemed highly pure and not interfering with the assay should be used. Finally, protein-related cell maturation is easier to assess early on rather than formulation- and quality attributes-related effects since these properties likely change over time. Of note, evaluation of molecules that target DCs as part of their MOA may not clearly distinguish the contribution of the MOA to cell maturation from concurrent PRRs interactions, potentially resulting in a combined activation effect stronger than that observed with common sensitivity controls. However, a strong DC maturation might indicate a high(er) potential to develop immunogenicity independently of the root cause for maturation.

DC activation assays, like most of the preclinical immunogenicity potential assessment tools, are relative in nature and require a strict context of use and adequate qualification for results to be interpreted appropriately. Due to its complexity and variability, a primary moDC-based assay may not be easily standardized into a kit format. As highlighted in section 2, results may only be interpreted within the range of the assay qualification using robust controls, an adequate number of technical and preparation replicates, and an experimental design fitting the intended use of the assay. Ideally, the root cause of a signal either due to the molecule/intended formulation or an impurity or CQA may need to be further elucidated as the impact on the project’s path may differ considerably and could potentially be achieved via molecular re-design, adaptations to the production/purification process, or formulation optimization (15). Besides the application of the DC maturation assay as a first line *in vitro* adjuvanticity and immunogenicity potential assessment tool, it is also broadly used to gain a mechanistic understanding of factors contributing to immunogenicity of novel molecule/formulation, and in this context often performed side by side with other non-clinical tools such as MAPPs and T cell proliferation assays.

A particular flavor of the assay is to assess whether the molecule’s aggregation state might play a role in DC activation. In this context, the assay should be applied to enhance the mechanistic understanding of the impact of aggregates in a qualitative rather than a quantitative manner or will otherwise need to be qualified using accepted standards to ensure it is fit to measure maturation in a statistically robust and reproducible manner. This poses a big challenge as concentrations of aggregates are typically very low in clinical drug products, and the assay may not be sensitive enough to reliably detect weak responses. At minimum, a low immunogenic sensitivity control, which optimally should reflect the structure of the tested molecule and be free of aggregates, is to be included in the assay to determine the threshold by which a positive signal can be measured and assessed. In the interpretation of the assay’s data, however, while a positive signal hints to a DC activation, the absence of a signal does not necessarily guarantee an absence of risk.

Besides a reproducible experimental setup, which enables the longitudinal performance evaluation of the assay over time, raw results need to be consistently analyzed using a fixed statistical model enabling a robust separation of negative/positive signals. In a secondary use of the assay, for example in conjunction with a MAPPs assay, DC maturation assays are carried out to interrogate whether cells are functional and to assess whether activation markers and HLA class II peptide presentation coincide (14). Accordingly, both assays are preferably performed side-by-side using moDCs isolated from the same donors since the DC maturation data can be used as a quality control to show that these cells are suitable for the MAPPs assay.

### 6.1 Future considerations

In the last few years, new therapeutic modalities have gained traction and using viral or retroviral vectors have held great promise for the treatment of patients. In particular, the use of adeno-associated virus (AAV) vectors has gained in popularity due to a lack of substantial clinical pathogenicity and the ease with which it can be customized to deliver a transgene into a variety of cells. In clinical development, however, mild to severe adverse events have been associated with host immune responses against AAV gene therapies, resulting in comprehensive evaluation of immunogenicity during nonclinical and clinical studies mandated by health authorities (39, 40). Similar to biotherapeutics, the immunogenicity risk of AAV vectors reflects a combination of product-, manufacturing process-, treatment-, and patient-related factors (40).

In humans, pre-existing immunity (including anti-AAV antibodies and reactive cytotoxic T cells induced by prior infections with wild type AAV’s) remains a major consideration, as it can partly limit the applicability of AAV-based gene therapies. Furthermore, the transgene-encoded proteins, whether secreted, presented on the cell surface, or localized intracellularly, may also induce an immune response (41). To date, most risk mitigation efforts have focused on optimizing the capsid amino acid sequence to avoid or minimize binding by pre-existing anti-capsid antibodies and activation of the complement system, which can in turn lead to activation of macrophages and DCs resulting in an enhanced humoral response. In addition, the vector genome has been under close scrutiny due to its increased risk to trigger innate immune responses via TLR2 and TLR9, leading to pro-inflammatory cytokine production and subsequent activation of adaptive immunity (42). Given this context, DC maturation assays offer a valuable approach to de-risk aspects of AAV-based therapeutics. Individual laboratories and CROs have begun adapting DC maturation assays to reflect the broader scope of immune activation by AAV vectors (28). However, the field still has some way to go in aligning with the established assay principles that encompass the diverse activation mechanisms underlying clinically observed immunogenic adverse reactions.

While considerably older, the field of nucleic acid therapeutics has also not progressed significantly in developing general assays dealing with immunogenicity risk assessment (43). Nevertheless, the successful development of two highly efficacious mRNA vaccines against COVID-19 underscored the potential of mRNA-based technology to deliberately activate the immune system. In a recent review, Sajeed Naeem et al. (44) note that the most pressing needs in the field are to enhance the delivery of the therapeutics to the target cells (including the use of carrier systems such as lipid nanoparticles or viral vectors) and to increase their stability in the native cellular environment. Immunogenicity was considered a lesser concern, possibly due to the powerful pre-clinical screening processes used in the development of nucleic acid therapeutics. However, in its latest report, the Oligonucleotide Safety Working Group (45) noted that information regarding immunogenicity of nucleic acid therapeutics remains limited, and that risk assessment in nonclinical studies is typically compound- and program-specific. Notably, the group further suggested that preclinical animal studies might provide information regarding intended or unintended effects related to ADA response characterization, while cautioning that the immune system of safety animal models may not accurately reflect the human scenario accurately. Therefore, these new modalities might benefit from non-clinical risk assessment, and, as with viral vectors, the nucleic acid therapeutics might require the set-up of specific DC maturation assays tailored towards CD8+ T cell activation.

To summarize, key elements such as cell source, cell culture conditions, reagents, and assay-specific defined criteria for baseline response and positivity can differ amongst laboratories. Therefore, the focus for harmonization lies in quality criteria at each state of the assay and the selection and use of proper controls, to allow meaningful data interpretation.
